# *In vivo* dynamic visualization and evaluation of collagen degradation utilizing NIR-II fluorescence imaging in mice models

**DOI:** 10.1093/rb/rbaf025

**Published:** 2025-04-11

**Authors:** Shunyao Li, Kai Xu, Huaixuan Sheng, Huizhu Li, Xiao Zhang, Chengxuan Yu, Haichen Hu, Xiner Du, Yunxia Li, Yu Dong, Jun Chen, Sijia Feng

**Affiliations:** Department of Sports Medicine, Huashan Hospital, Fudan University, Shanghai 200040, China; Sports Medicine Institute of Fudan University, Shanghai 200040, China; Department of Sports Medicine, Huashan Hospital, Fudan University, Shanghai 200040, China; Sports Medicine Institute of Fudan University, Shanghai 200040, China; Department of Sports Medicine, Huashan Hospital, Fudan University, Shanghai 200040, China; Sports Medicine Institute of Fudan University, Shanghai 200040, China; Department of Sports Medicine, Huashan Hospital, Fudan University, Shanghai 200040, China; Sports Medicine Institute of Fudan University, Shanghai 200040, China; Department of Sports Medicine, Huashan Hospital, Fudan University, Shanghai 200040, China; Sports Medicine Institute of Fudan University, Shanghai 200040, China; Department of Sports Medicine, Huashan Hospital, Fudan University, Shanghai 200040, China; Sports Medicine Institute of Fudan University, Shanghai 200040, China; Department of Sports Medicine, Huashan Hospital, Fudan University, Shanghai 200040, China; Sports Medicine Institute of Fudan University, Shanghai 200040, China; Department of Sports Medicine, Huashan Hospital, Fudan University, Shanghai 200040, China; Sports Medicine Institute of Fudan University, Shanghai 200040, China; Department of Sports Medicine, Huashan Hospital, Fudan University, Shanghai 200040, China; Sports Medicine Institute of Fudan University, Shanghai 200040, China; Department of Sports Medicine, Huashan Hospital, Fudan University, Shanghai 200040, China; Sports Medicine Institute of Fudan University, Shanghai 200040, China; Department of Sports Medicine, Huashan Hospital, Fudan University, Shanghai 200040, China; Sports Medicine Institute of Fudan University, Shanghai 200040, China; Department of Sports Medicine, Huashan Hospital, Fudan University, Shanghai 200040, China; Sports Medicine Institute of Fudan University, Shanghai 200040, China

**Keywords:** collagen, degradation, NIR-II fluorescence imaging, *in vivo*, cartilage defect, crosslinking degree

## Abstract

Collagen-based biomaterials are gaining prominence in tissue engineering, attributed to their remarkable biocompatibility, inherent biodegradability, and unparalleled capacity to facilitate tissue repair and regeneration. However, the ability to dynamically visualize and quantitatively assess collagen degradation *in vivo* remains a critical challenge, hindering the development of optimized biomaterials for clinical applications. To address this, a novel approach was developed to monitor the injury microenvironment by conjugating second near-infrared quantum dots with solid collagen. This live imaging system offered high-resolution, real-time tracking of collagen degradation both *in vitro* and *in vivo*, enabling a deeper understanding of the degradation behavior under various conditions. This system was applied to mouse models with different cartilage defects, including critical-sized defect (CSD), minor defect (Minor) and sham surgery (Sham) groups for a 28-day *in vivo* monitoring. Among them, the CSD group exhibited the fastest and most stable collagen degradation, indicating that the degradation rate was closely linked to the severity of the injury. Transcriptomic analysis further identified key signaling pathways that might drive rapid collagen degradation by promoting collagenase activity and tissue remodeling in cartilage defect conditions. In summary, our study provided valuable insights into the mechanisms of collagen degradation under different injury conditions, contributing to innovative strategies for designing collagen-related biomaterials in the future.

## Graphical abstract

**Figure:**
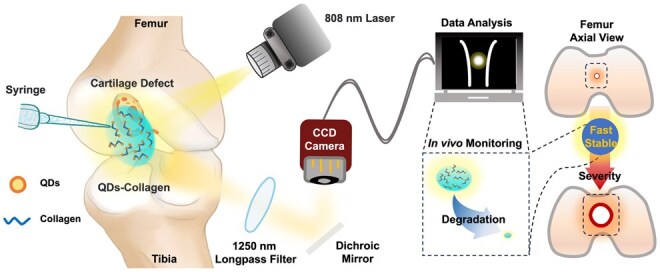

## Introduction

Collagen, a biocompatible and biodegradable extracellular matrix protein, possesses a unique triple-helical conformation that endows it with remarkable mechanical strength and flexibility [1–5]. The biocompatibility, minimal immunogenicity and precisely controllable degradation kinetics of collagen have positioned it as a premier candidate for tissue repair applications, where it is instrumental in enhancing cell adhesion, migration and proliferation [1, 2, 6–10]. Collagen-based biomaterials have been engineered to fabricate scaffolds with tailored pore architectures, mechanical properties and degradation profiles to meet the specific demands of tissue engineering endeavors [8–14]. However, the extant research on collagen as a novel biomaterial has yielded inconsistent and unpredictable outcomes [1, 12, 13]. Therefore, a comprehensive evaluation of collagen in tissue injury is currently in urgent need.

The majority of evaluations pertaining to the efficacy of collagen-based biomaterials in tissue injury have been conducted under *in vitro* conditions [15–21]. Subsequent evaluations have been extended to animal models to ascertain the reparative potential of implanted collagen constructs [15, 18, 19, 22–24]. Nevertheless, challenges persist in the accurate quantification of collagen-based biomaterials, particularly with respect to the degradation rate of collagen, which is intricately linked to the kinetics of tissue formation [25–29]. The complexity and variability of *in vivo* environments have made the spatiotemporal dynamics of collagen degradation difficult to monitor, leading to an imprecise understanding of the temporal progression, mechanisms, and biomechanical alterations induced by collagen, thus casting doubt on its clinical utility [3, 30–34]. Hence, there is an imperative for *in vivo* studies that can provide objective and reliable data on collagen degradation processes.

Fluorescence imaging, particularly in the second near-infrared (IR) window (NIR-II, 900–1700 nm), has emerged as a significant advancement in biomedical engineering for the *in vivo* evaluation of biological phenomena [30, 35–43]. NIR-II fluorescence imaging offers enhanced diffraction-limited resolution, increased tissue penetration, reduced scattering and diminished background interference, thereby facilitating the precise mapping of collagen’s spatiotemporal distribution [44–50]. The development of protein-coated, stable and sensitive lead sulfide quantum dots (QDs) for *in vivo* labeling of collagen has been successfully demonstrated, enabling long-term fluorescence imaging and offering a clear view of the collagen degradation process across various anatomical regions in animal models. This approach holds the promise of yielding dynamic, high-fidelity data on collagen degradation kinetics, including specific timelines, rates and stability [30]. Consequently, NIR-II fluorescence imaging is regarded as a promising modality for the real-time, dynamic, and *in vivo* visualization and evaluation of collagen degradation.

In this study, an NIR-II fluorescence imaging system based on QDs was developed to visualize the dynamic degradation of collagen and elucidate the underlying mechanisms. Initially, collagen was labeled with QDs and subjected to *in vitro* testing to ascertain its physical and optical characteristics. Subsequently, a subcutaneous injection model was established to correlate the fluorescence trends with the actual degradation process of collagen. Further, articular cartilage injuries including minor defect (Minor) and critical-sized defect (CSD) were induced in mice models respectively and the degradation process of collagen was monitored over a 28-day period, compared with a sham surgery (Sham) group. This investigation holds the potential to unravel the complex patterns of collagen degradation during articular cartilage injury through dynamic *in vivo* imaging. This advancement is of paramount importance as it enhances our collective comprehension of collagen degradation mechanisms, which are fundamental to the pathophysiology of tissue injury and can lead to innovative strategies in the realm of tissue engineering and regenerative medicine.

## Methods

### Reagents and instrumentation

Lead (II) acetate trihydrate [Pb(CH_3_CO_2_)_2_·3H_2_O, ≥99%], sodium sulfide (Na_2_S, ≥90%) and ribonuclease A (RNase A, >70 U/mg) were purchased from J&K Scientific (China) and Sigma-Aldrich (USA), respectively. Sodium hydroxide (NaOH, ≥95%) was obtained from Shanghai Macklin Biochemical Co., Ltd (China). 3M Vetbond Tissue Adhesive was purchased from Shanghai Yuyan Instruments (China). Deionized water was used as the working solution. Microwave synthesis was performed using a Discover SP microwave reactor (USA). NIR-II fluorescence imaging was conducted using an 808 nm excitation source provided by two fiber-coupled lasers with a power of 100 mW/cm^2^. The emitted light was filtered through a 1250 nm bandpass filter and collimated with a 50 mm focal length NIR-II lens from Goldeye G-130 TEC1 Allied Vision (China). Detection was performed using a liquid nitrogen-cooled InGaAs camera from Teledyne Princeton Instruments Trenton (USA).

### Preparation of liquid and solid QD-labeled collagen

Highly fluorescent lead sulfide (PbS) QDs encapsulated with RNase A were synthesized using a previously established method [49]. Briefly, 500 µl of 10 mM Pb(CH_3_CO_2_)_2_ and 500 µl of 50 mg/ml RNase A were mixed, and the pH was adjusted to 9-10 with 1 M NaOH. Then, 50 µl of 10 mM Na_2_S was added. The mixture was placed in a microwave reactor at 70°C for 30 s, resulting in a dark brown transparent liquid. Mouse tail collagen type I from Solarbio Science & Technology Co., Ltd (China) was first dissolved in 0.1 M MES (2-[N-morpholino] ethanesulfonic acid) buffer and mixed with varying amounts of crosslinkers at room temperature for 2 h, according to the previous protocol [51]. The liquid collagen was conjugated with QDs for 2 h using a mass ratio of 1:1.15:2.76 for liquid collagen, EDC and NHS to achieve a 100% crosslinking degree (100%C-Q). Excess liquid was removed by ultrafiltration at 4°C using a 10 kDa molecular weight at 3000 rpm for 15 min. The purified collagen–QDs conjugates were used for subsequent structural characterization and *in vivo* implantation experiments. For 10% and 50% crosslinking degrees, the proportions were adjusted accordingly. To prepare solid collagen, 1 ml of collagen was mixed with 12 µl of 0.1 M NaOH and incubated at 4°C for 2 h. Then, 100 µl of 10× PBS was added, and the mixture was incubated at room temperature for 20 min to promote solidification, following the same crosslinking procedure as for the liquid collagen.

### Characterization of QD-labeled collagen

The freshly prepared liquid QD-labeled collagen was stored at 4°C. The ultraviolet-visible (UV-vis) spectrum and IR spectra of the QD-labeled collagen were assessed. IR spectroscopy was performed with a resolution of 4000 cm^−1^, utilizing 32 scans for both sample and background, and a DTGS KBr detector. UV-vis spectroscopy was conducted over a wavelength range of 200–900 nm. For scanning electron microscopy (SEM), the solid collagen samples were examined at an acceleration voltage of 5.0 kV, with a working distance of 0.6 mm and magnifications ranging from ×2000 to ×30 000, to assess surface morphology and structural features.

### *In vitro* observation of solid QD-labeled collagen degradation

Type I collagenase from Millipore (USA) was prepared as a working solution of 4 mg/ml. To prepare the solution, 400 mg of collagen type I powder was dissolved in 100 ml DMEM/F12 (Millipore) and filtered using a 0.22 μm Stericup^®^-GP filter unit to ensure sterility. Collagen samples were dried at 37°C for 30 min to remove moisture and then weighed using an analytical balance to record the initial mass. The dried samples were placed in 12-well plates, with 3 mm^3^ of collagen in separate wells. Each sample was fully immersed in 0.5 ml of the collagenase solution. The plates were incubated at 37°C for 7 days, during which the mass percentage and NIR-II photoluminescence (PL) intensity were monitored. At specified intervals, samples were removed, dried again at 37°C for 30 min, and reweighed to determine the final mass for degradation analysis. The procedure was adapted from previous research [16, 17, 21]. Postoperative analgesia was administered as needed. The average mass percentage was calculated using the following formula: $\mathrm{Mass}\;\mathrm{percentage}=\frac{\text{Final}\;\text{mass}}{\text{Original}\;\text{mass}}$. Exponential decay model (*y* = span·e-kx+plateau) was utilized for curve fitting by GraphPad Prism 9.3.1 [52, 53], and *k*, as PL intensity change rate, was calculated as follows in this work:


$$k=\frac{{\ln(\mathrm{relative}\;\mathrm{PL}\;\mathrm{intensity}}^{-1})}{\mathrm{days}}.$$

### Establishment of mice models with subcutaneous collagen implantation

Eight-week-old female Institute of Cancer Research (ICR) mice, weighing 25–35 g, were obtained from Shanghai Jiesijie Laboratory Animal Co., Ltd. All procedures were performed in accordance with the guidelines approved by the Animal Care Committee of Fudan University (202409059S). Mice were anesthetized using isoflurane at a flow rate of 1.5 l/min with oxygen at 0.2 l/min. The dorsal region was shaved and disinfected with 70% ethanol. A small, 1 cm longitudinal incision was made in the skin using sterile scissors. The underlying subcutaneous tissue was carefully bluntly dissected using forceps to create a small pocket. Collagen samples (3 mm^3^) were implanted into the subcutaneous space. Care was taken to ensure that the collagen was properly positioned within the pocket to avoid movement or extrusion. Following the implantation, the incision site was closed using 6-0 absorbable sutures from Ethicon (China). The mice were monitored postoperatively until they fully recovered from anesthesia. No additional interventions were required, and the mice were housed under standard conditions for further observation.

### Establishment of mice models of articular cartilage defects with collagen implantation

Mice were anesthetized with isoflurane (1.5 l/min) and oxygen (0.2 l/min). After shaving and disinfecting the left hind leg, an incision was made from the tibia to the quadriceps muscle. The knee joint capsule was cut, and the patella was dislocated by extending the joint, allowing full flexion to expose the trochlear groove. A full-thickness CSD with a diameter of 1 mm was created by scratching the articular surface with a 25-G needle, based on previous studies [54–56]. For the Minor group, a 29-G needle was used to create a defect with a diameter of 150 μm, also based on established methods from these references. The Sham group underwent no further intervention. Subsequently, collagen was thoroughly packed into the defect site and secured using 3M Vetbond Tissue Adhesive for both the CSD and Minor groups. After implantation, the site was left undisturbed for 15–30 min to allow stabilization. The joint was then irrigated with saline and closed with 6-0 Vicryl from Ethicon (China). The skin was sutured using 6-0 Ethilon (Ethicon). Mice were euthanized at 28 days post-surgery by cervical dislocation under isoflurane/O_2_ anesthesia.

### *In vivo* observation of solid QD-labeled collagen degradation in mice models based on NIR-II fluorescence imaging

Mice were first anesthetized using isoflurane at a flow rate of 1.5 l/min while oxygen at a flow rate of 0.2 l/min was administered. Then NIR-II images were obtained at 1 h and 1, 2, 3, 7, 14, 21, 28 and 35 days after injection as appropriate (28 days for joints, 35 days for subcutaneous tissue). The PL intensity and fluorescence area of the region of interest from NIR-II images were measured using ImageJ. The degradation rate *k* was calculated as mentioned above. PL intensity data were analyzed using Pearson’s correlation coefficients to evaluate the variation of collagen degradation over time. Correlation values (*r*) were calculated for each time point across the different defect groups (CSD, Minor, Sham). Density plots were generated to display the distribution of PL intensities, and scatter plots were used to visualize the correlation trends across time points.

### Histological analysis

The subcutaneous tissue in subcutaneous collagen implantation was harvested to perform histological analysis. The whole layer of skin with a size about 0.5 × 0.2 cm was collected from subcutaneously injected points. The major organs including cerebrum, lung, heart, stomach, kidney, spleen, liver, colon and intestine were harvested and the hematoxylin-eosin (H&E) staining histological analysis of major organs was applied.

### Transcriptomic analysis

All mice with femoral intercondylar notch cartilage defects, which received collagen implants, were euthanized at 0 and 7 days post-implantation, and their cartilage tissues were collected for transcriptomic analysis. RNA-seq analysis, enrichment of mRNA, fragmentation, reverse transcription, library construction were performed by Personalbio Technology Co., Ltd (Shanghai, China). The thresholds for differentially expressed genes (DESeq) were *P *<* *0.05 and fold change ≥1. DESeq was subjected to function and signaling pathway enrichment analysis using KEGG (Kyoto Encyclopedia of Genes and Genomes) databases to identify the underlying signaling pathways that potentially drove collagen degradation by cartilage defect.

### Statistics analysis

Correlation graphs were analyzed using the *R* language package. The relationship between PL intensity and mass percentage was evaluated using Pearson’s correlation coefficient (*r*). The correlation coefficient quantified the strength and direction of the linear relationship between these variables, with statistical significance determined by *P*-values. Curve fitting was conducted with GraphPad Prism (version 9.3.1), applying an exponential decay model to describe the degradation pattern of collagen samples. The correlation coefficients (Corr) were calculated between fluorescence intensity values at different time intervals to examine the relationship between degradation progression and time. Density plots were generated to visualize the distribution of fluorescence intensity across the groups, highlighting the variation in degradation rates. The *P*-values are calculated based on the hypergeometric distribution, and the significant pathway (*P *<* *0.05) was used for further study. Significant correlations and model fits were indicated with asterisks in the figures: ∗*P *<* *0.05, ∗∗*P *<* *0.01 and ∗∗∗*P *<* *0.001.

## Results and discussion

**Figure 1.**
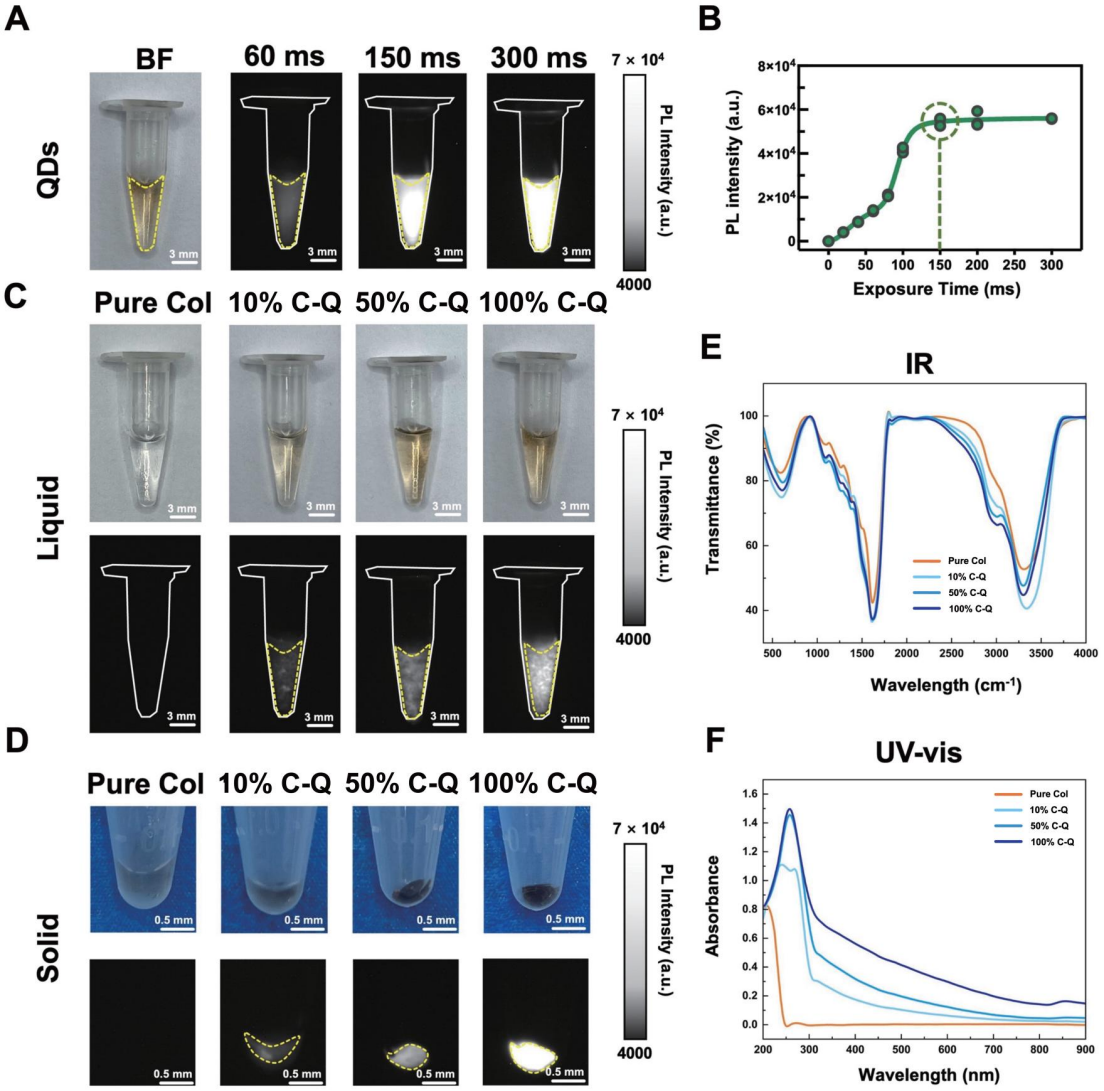
Characterization of QD-labeled collagen. (**A**) BF and NIR-II images of QDs at different exposure times (60, 150, 300 ms). (**B**) PL intensity measured from NIR-II images at different exposure times. BF and NIR-II images of QDs labeled (**C**) liquid and (**D**) solid collagen at different crosslinking degrees (10% C-Q, 50% C-Q, 100% C-Q) compared with pure collagen (pure col). (**E**, **F**) IR spectra and UV absorbance spectra of liquid collagen (Pure Col, 10% C-Q, 50% C-Q, 100% C-Q).

The *in vitro* stability and fluorescence properties of both liquid and solid collagen labeled with NIR-II QDs (C-Q) were first investigated. As shown in Figure 1A, the original QDs appeared as transparent yellow in color in an Eppendorf tube (EP tube). Then, NIR-II fluorescence imaging was conducted for QDs at different exposure times from 0 to 300 ms. The QDs showed optimal fluorescence intensity from exposure time of 150 ms, after which the intensity plateaued, indicating the ideal exposure time for collagen monitoring (Figure 1B). Bright-field (BF) images of liquid collagen with varying crosslinking degrees (10%, 50%, 100%) showed a darker appearance upon QDs labeling, while the corresponding NIR-II fluorescence images confirmed the distribution of QD-labeled collagen within the liquid phase, in accordance with the BF images (Figure 1C). Similarly, BF and NIR-II fluorescence images of solid collagen at different crosslinking degrees in Figure 1D demonstrated a matching spatial distribution of labeled collagen in the solid state, suggesting that the NIR-II fluorescence signal distribution could represent the distribution of collagen. The successful labeling of collagen was further observed under microscopic examination (see Supplementary Figure S1), while the SEM results confirmed that the QD labeling had no effect on the surface morphology of collagen (see Supplementary Figure S2).

In addition, IR spectra (Figure 1E) and UV-vis spectrum (Figure 1F) were obtained for liquid collagen with varying degrees of crosslinking. The IR spectra of all samples displayed similar curves, indicating a similar absorption in IR wavelength. While the UV-vis spectrum of C-Q at different crosslinking degrees exhibited a peak at around 280 nm compared with pure collagen peaked around 200 nm. This peak was considered the characteristic peak of the protein enveloping QDs, suggesting the successful labeling of collagen by QDs. In summary, 150 ms was identified as the optimal exposure time for *in vitro* collagen observation, and 100% crosslinking degree resulted in the highest PL intensity and optical property, which could be further chosen to be adopted in the *in vivo* observation.

**Figure 2.**
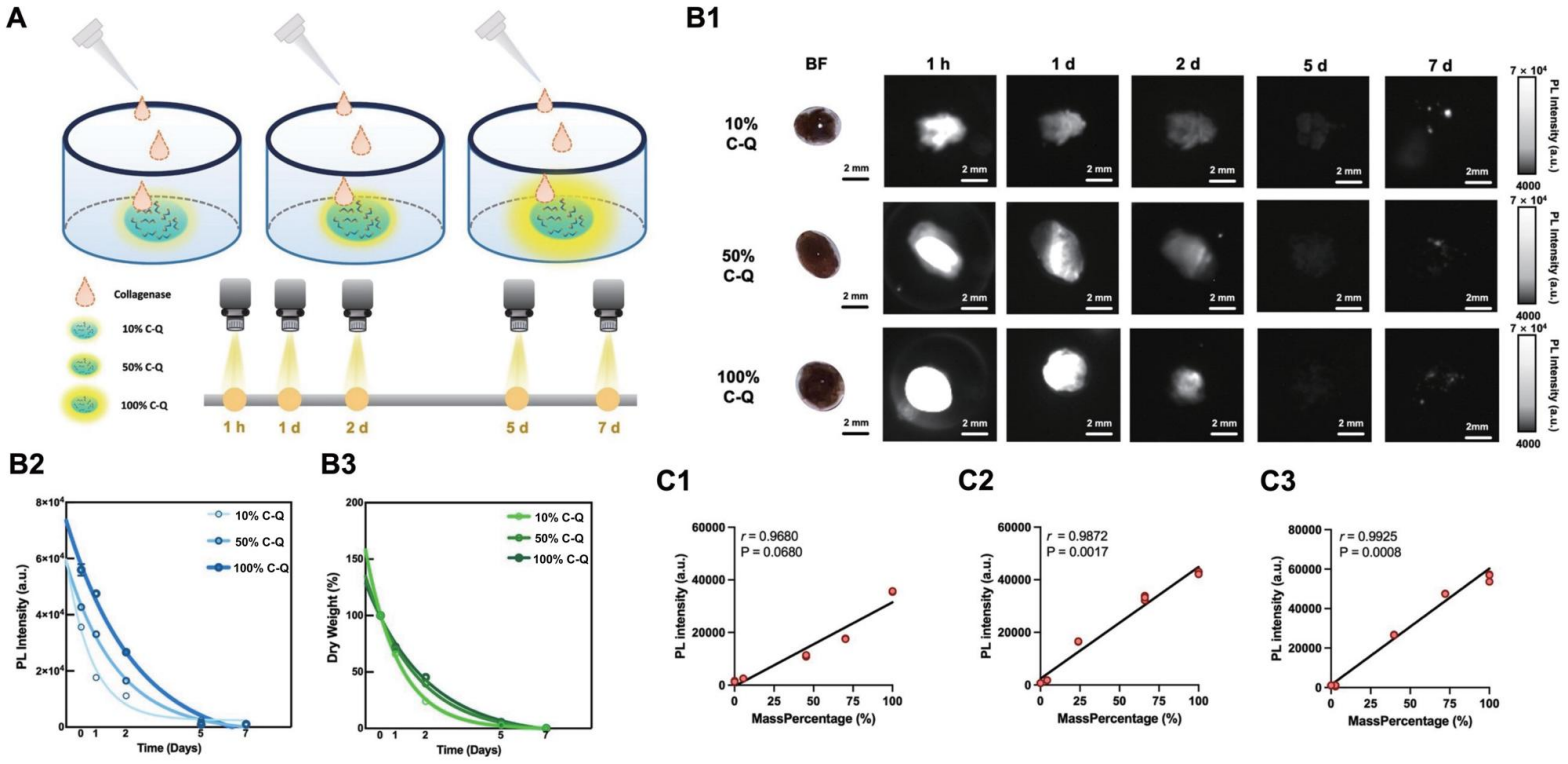
*In vitro* observation of solid collagen degradation. (**A**) Schematic illustration of observation of solid collagen degradation in a 12-well plate utilizing NIR-II fluorescence imaging in a time course (1 h and 1, 2, 5 and 7 days). (**B1**) BF and NIR-II images of QD-labeled solid collagen at different time points. (**B2**) PL intensity measured from NIR-II images in (**B1**). (**B3**) Dry weight of solid collagen measured at each time point. (**C1**–**C3**) Pearson correlation analysis between PL intensity and mass percentage of collagen samples at different crosslinking degrees (10%, 50%, 100%) across time points.

The degradation of solid collagen labeled with NIR-II QDs at varying crosslinking degrees (10%, 50%, 100%) was further observed and analyzed *in vitro* in 12-well plates (Figure 2A). The fluorescence showed strongest signals at 1 h and gradually diminished over 7 days (Figure 2B1). As shown in Figure 2B2, the PL intensity of the solid collagen at different crosslinking degrees decreased in a similar trend. The dry weight loss also depicted a decreasing trend among the groups, indicating that the fluorescence signal disappearance precisely represented the collagen degradation process (Figure 2B3). Furthermore, the correlation analysis between fluorescence changes and MassPercentage changes (Figure 2C1–C3) indicated that strong positive correlations were observed across the three crosslinking degrees. Compared with the 10% C-Q group (Figure 2C1), both the 50% C-Q group (*r* = 0.9872, *P *=* *0.0017) and the 100% C-Q group (*r* = 0.9925, *P *=* *0.0008) showed statistically significant correlation (Figure 2C2–C3). It was indicated that the decrease of PL intensity could excellently reflect the process of collagen degradation especially at a crosslinking degree of 100%, which was selected for subsequent *in vivo* implantation experiments based on the reliable correlation.

**Figure 3.**
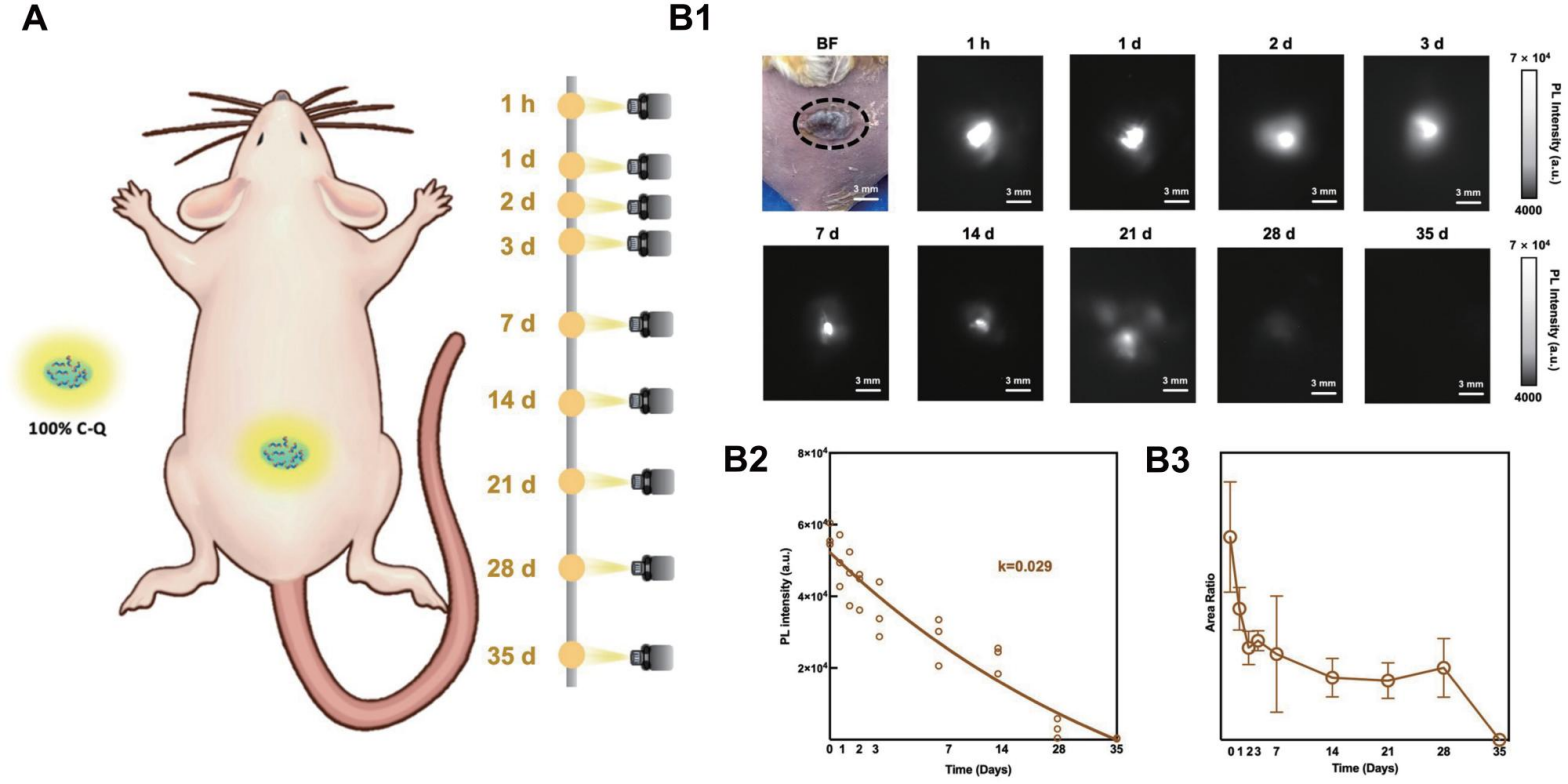
*In vivo* observation of solid collagen degradation. (**A**) Schematic illustration of observation of solid collagen degradation implanted subcutaneously at the back of mice (*n* = 3) utilizing NIR-II fluorescence imaging in a time course (1 h and 1, 2, 3, 7, 14, 21, 28 and 35 days). (**B1**) BF (1 h) and NIR-II images of QD-labeled solid collagen in a mouse model at different time points. (**B2**) PL intensity and (**B3**) fluorescence area measured from NIR-II images in (**B1**).

Based on the *in vitro* experiments, solid collagen labeled with QDs was implanted subcutaneously at the back of mice and monitored over time using NIR-II fluorescence imaging at an exposure time of 300 ms (Figure 3A and Supplementary Figure S3). The fluorescence signals from the implanted collagen showed a continuous decrease from 1 h to 35 days post-implantation, indicating progressing *in vivo* degradation of the collagen over the 35-day period (Figure 3B1). Quantitative analysis further confirmed this trend, showing a consistent decline in PL intensity and fluorescence area over time (Figure 3B2–B3). The degradation coefficient *k* for this process was calculated as 0.029, reflecting the rate of collagen degradation subcutaneously. At 35 days post-implantation when the fluorescence completely dissipated, histological analysis of the subcutaneous tissue confirmed that the collagen had fully degraded (see Supplementary Figure S4). Furthermore, in comparison with the pure collagen implantation group and the healthy group, subcutaneous implantation of 100% C-Q induced no obvious immune or inflammatory responses (see Supplementary Figure S4). In short, NIR-II fluorescence imaging allowed for real-time monitoring of subcutaneous solid collagen degradation over 35 days, showing its high sensitivity in tracking *in vivo* collagen-based biomaterial breakdown. Besides, the complete degradation of collagen, without triggering immune or inflammatory responses, further underscores the biocompatibility of the implanted QD-labeled material.

**Figure 4.**
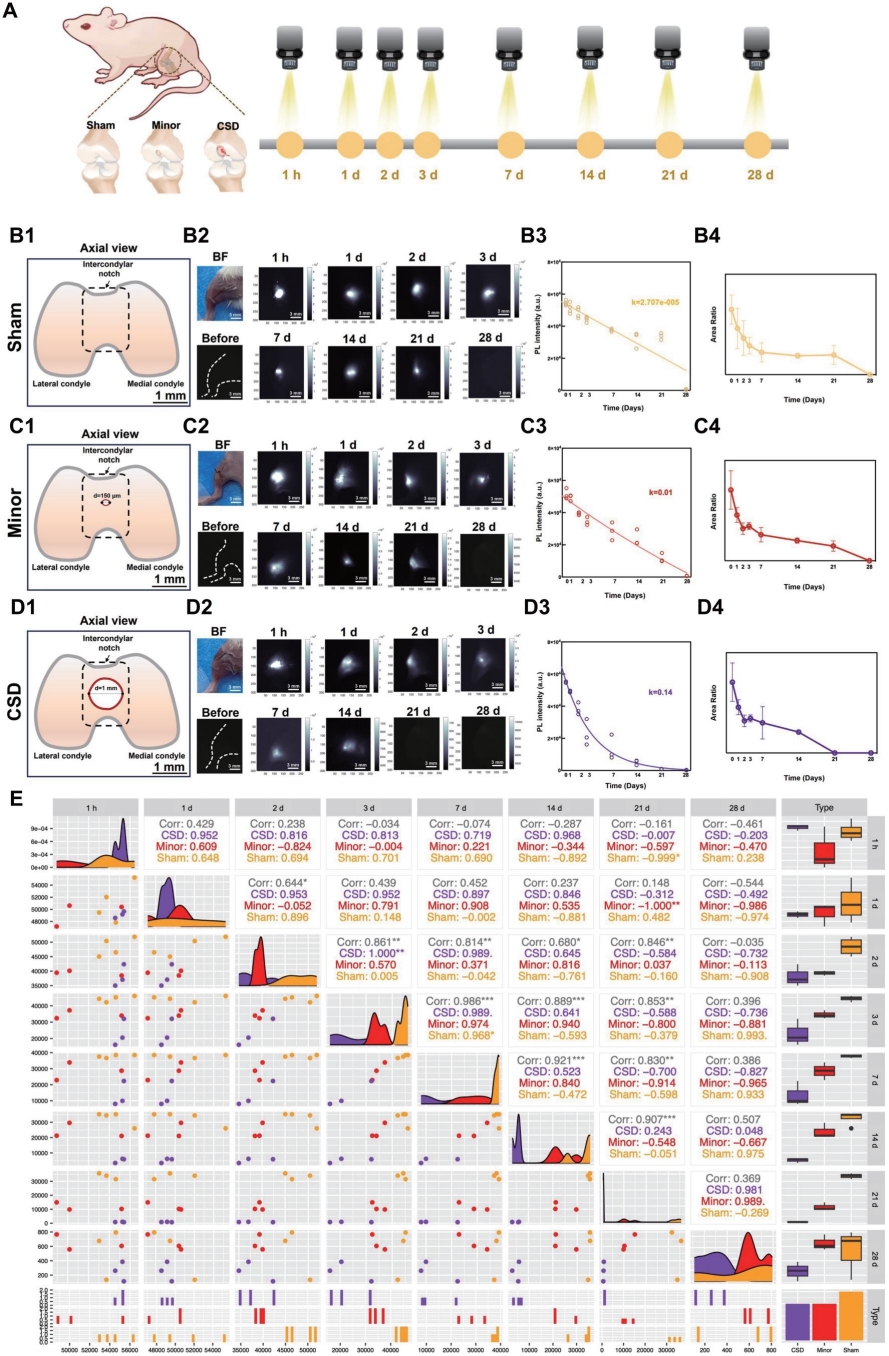
*In vivo* observation of solid collagen degradation in articular cartilage defect mouse models. (**A**) Schematic illustration of observation of solid collagen degradation implanted intra-articularly in the joint of a mouse utilizing NIR-II fluorescence imaging in a time course (1 h and 1, 2, 3, 7, 14, 21, 28 days). Schematics showing axial view of (**C1**) Minor group and (**D1**) CSD group created in the articular cartilage of mice models compared with the (**B1**) Sham group. BF and NIR-II images of intra-articularly implanted collagen in mice models of (**C2**) the Minor group and (**D2**) the CSD group compared with (**B2**) the Sham group in a time course. (**B3**, **C3**, **D3**) PL intensity and (**B4**, **C4**, **D4**) fluorescence area measured from NIR-II images in (**B2**, **C2**, **D2**). Collagen degradation in different groups was demonstrated in the correlation plot. Comprehensive overview (**E**) of the fluorescence intensity correlations and degradation stability across different time points for the CSD, Minor and Sham groups.

To explore the specific process of solid collagen degradation during tissue injury, mice models of small and large articular cartilage defects were established along with the Sham group, with the NIR-II QD-labeled solid collagen (100% C-Q) implanted intra-articularly and observed over a time course (1 h and 1, 2, 3, 7, 14, 21 and 28 days) (Figure 4A). Cartilage defects with diameters of 0.15 mm in the Minor group and 1 mm in the CSD group were created in the center of the intercondylar notch, compared to the Sham group (Figure 4B1, C1, D1 and Supplementary Figure S5). Following implantation of the solid C-Q into the knee joints of mice models, continuous dynamic observation was performed utilizing NIR-II fluorescence imaging at an exposure time of 200 ms (see Supplementary Figure S6).

The fluorescence signals in the knee joints consistently decreased over time across all groups (Figure 4B2, C2, D2). The PL intensity measured from NIR-II fluorescence images was fitted into curves (Figure 4B3, C3, D3), and the degradation coefficients (*k*) were calculated for each group. The fluorescence area analysis also revealed a consistent decrease over time (Figure 4B4, C4, D4). The Sham group exhibited the lowest degradation coefficient of *k* = 2.707e−005 (Figure 4B3). Meanwhile, the Minor group had a degradation coefficient of *k* = 0.01 (Figure 4C3). While the CSD group exhibited the highest degradation coefficient of *k* = 0.14 (Figure 4D3), indicating the fastest degradation rate among the groups.

The stability of degradation in each group was determined using the correlation coefficient, which quantified the linear relationship between the measured fluorescence signals over time [57] (Figure 4E). The Minor group’s correlation coefficients showed fluctuating across time points, reflecting an unstable and inconsistent degradation process, followed by a significant drop in degradation rates and high fluctuations at 28 days post-injury. Notably, the CSD group exhibited consistently high correlation coefficients during the early phase (1 h to 3 days), though a gradual decrease in correlation was observed from 14 days onward, with some fluctuations at 28 days post-injury.

The local microenvironment following cartilage injury may significantly influence collagen degradation. Cartilage injury often induces inflammatory responses, oxidative stress and matrix metalloproteinases (MMPs) secretion [58, 59]. Several studies have reported that the primary factor in collagen degradation is the presence of collagenases, which are specialized enzymes that cleave the collagen triple helix at specific sites, especially MMPs [20, 59, 60]. Moreover, mechanical stress at the cartilage injury site may further exacerbate collagen degradation through increased inflammatory responses and enzymatic activity, which is likely to contribute to the distinct pattern observed among groups, with larger defects promoting a more sustained inflammatory microenvironment, leading to faster and more stable degradation [60]. In summary, our data demonstrated that collagen degradation occurred more rapidly and stably in company with the increase in the severity of injury.

**Figure 5.**
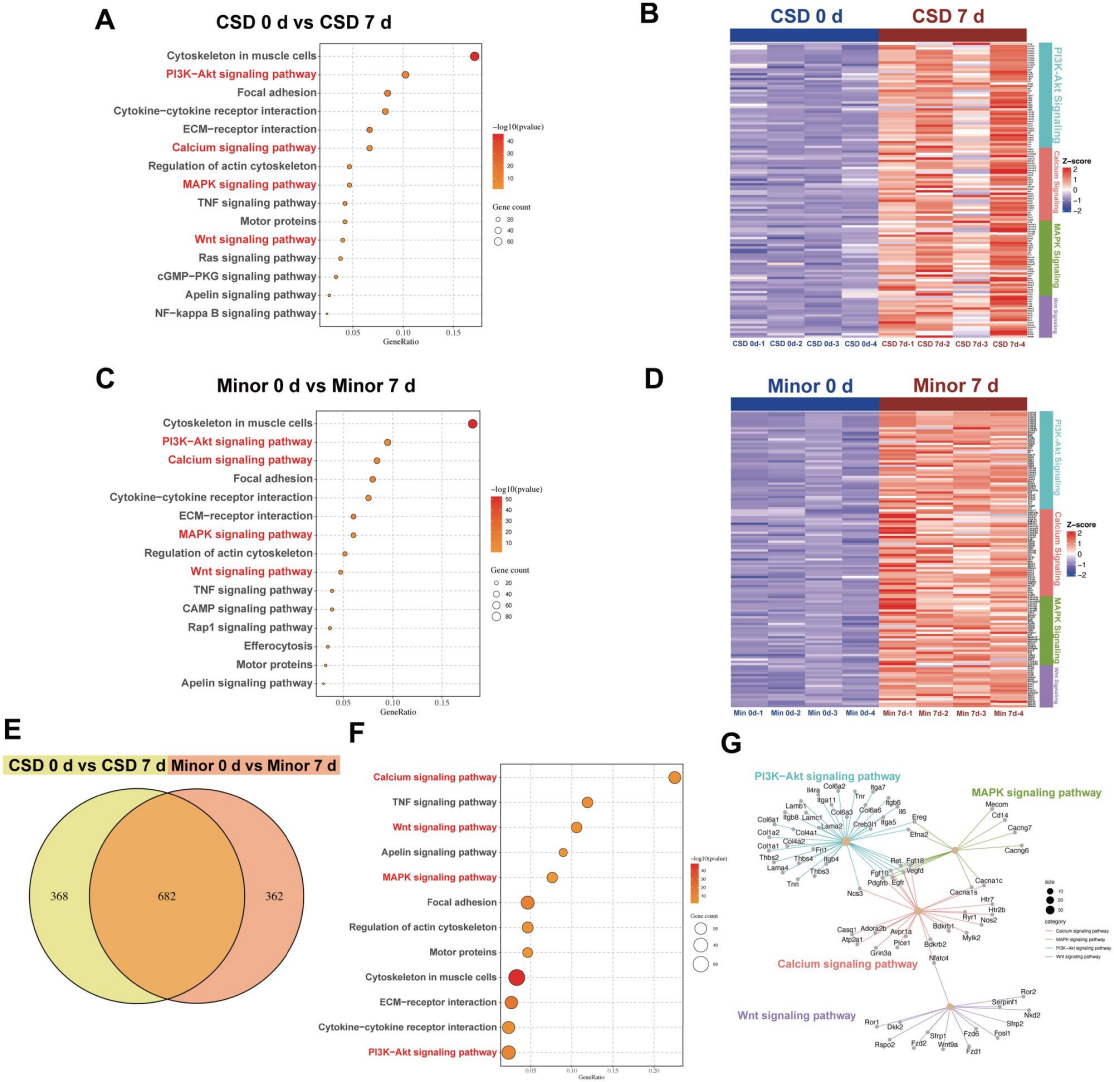
Transcriptomic analysis. KEGG pathway enrichment and heatmap for (**A** and **B**) the CSD group day 0 versus CSD group day 7 and (**C** and **D**) the Minor group day 0 versus minor group day 7. (**E**) Venn diagram of differentially expressed genes (DEGs) between the CSD group day 0 vs CSD group day 7 and the Minor group day 0 vs Minor group day 7. (**F**) Top enriched pathways associated with DEGs from both comparisons. (**G**) Network analysis.

Building upon these findings, transcriptomic analysis was performed in order to gain deeper insights into the underlying mechanisms driving collagen degradation (Figure 5). After implanting collagen in different groups, the cartilage tissue was harvested for transcriptomic profiling. The KEGG analysis revealed that the activation of key signaling pathways, such as PI3K-Akt, MAPK, Calcium and Wnt, drove the rapid collagen degradation observed from 0 to 7 days in both the CSD and Minor groups (Figure 5A–D). The upregulation of these pathways underscored their central role in regulating collagenase activity and tissue remodeling. Next, the common genes were filtered to further analyze their roles in collagen degradation. The results showed a high degree of similarity between the two injury models, suggesting a common mechanism for collagen degradation (Figure 5E–F). Further, the interplay was highlighted between these pathways, emphasizing their coordinated activation as critical to the degradation and repair processes following different degrees of injuries (Figure 5G).

Specifically, the PI3K-Akt signaling pathway exhibited enhanced activation by day 7. It has been shown to influence the expression of MMPs, particularly in the context of inflammation and tissue remodeling [61–64]. Activation of the Wnt pathway can lead to the stimulation of MAPK signaling cascades, which further modulate MMPs activity, demonstrating a significant increase in activity at 7 days and potentially affects the collagen degradation [65]. Calcium signaling, which showed substantial enrichment, is also implicated in collagen degradation through its influence on various signaling pathways. The non-canonical Wnt/Ca^2+^ pathway activates intracellular calcium release, which can stimulate calcineurin and other calcium-dependent kinases that regulate MMPs activity [66–68]. Besides, changes in intracellular calcium ion concentrations can directly influence the activity of collagenases, facilitating the collagen degradation [68, 69]. Thus, it was implied that the upregulation of PI3K-Akt, MAPK, Calcium and Wnt signaling pathways might lead to the collagen degradation by activating various mechanisms after cartilage defect, thereby contributing to the remodeling of the extracellular matrix and promoting healing.

To further assess the possible toxic impact of QD-labeled collagen on inner organs, histological analysis was performed on major organs including the colon, lung, liver, cerebrum, spleen, kidney, stomach, intestine and heart were harvested from the CSD, Minor and Sham groups, along with the blank group. The analysis revealed no obvious signs of morphological alterations or inflammation in the organs of all the groups compared with the blank group (see Supplementary Figure S7), proving the biosafety of QD-labeled collagen. These findings were consistent with previous studies proving low biotoxicity of both pure QDs and QD-labeled collagen [50].

## Conclusion

In this study, solid collagen was successfully labeled with NIR-II QDs, revealing correlations between PL intensity and collagen weight at varying degrees of crosslinking. The NIR-II monitoring approach effectively enabled real-time monitoring of microenvironmental variations in both normal physiological conditions and various injury scenarios *in vivo*. Notably, collagen degradation rates varied significantly with injury severity: the Sham group exhibited the slowest degradation (*k* = 2.707e−005), while the Minor group showed intermediate degradation (*k* = 0.01), and the CSD group demonstrated the fastest rate (*k* = 0.14). Degradation stability also differed, with the Minor group showing the most variability, particularly at 7 and 28 days post-injury, whereas the CSD group exhibited a consistent degradation pattern, especially from 1 h to 3 days. Additionally, the transcriptomic analysis revealed the key signaling pathways whose activation might lead to the rapid collagen degradation under cartilage defects. By capturing both the rate and stability of collagen degradation *in vivo*, this method allowed for real-time monitoring of collagen changes in response to the injury microenvironment, thus paving the way for innovative strategies in tissue engineering and regenerative medicine.

## Supplementary Material

rbaf025_Supplementary_Data

## Data Availability

The data that support the findings of this study are available from the corresponding author upon reasonable request.
