# Supplementary material for: In vivo dynamic visualization and evaluation of collagen degradation utilizing NIR-II fluorescence imaging in mice models
Source: Regen Biomater. 2025 Apr 11;12:rbaf025. doi: 10.1093/rb/rbaf025 (PMC12094926; doi:10.1093/rb/rbaf025)
Supplement: rbaf025_Supplementary_Data [file rbaf025_supplementary_data.docx]

**Supporting Information**

***In vivo* dynamic visualization and evaluation of collagen degradation utilizing NIR-II fluorescence imaging in mice models**

Shunyao Li^1#^, Kai Xu^1#^, Huaixuan Sheng^1#^, Huizhu Li^1^, Xiao Zhang^1^, Chengxuan Yu^1^, Haichen Hu^1^, Xiner Du^1^, Yunxia Li^1^, Yu Dong^1*^, Jun Chen^1*^, Sijia Feng^1*^

^1^Department of Sports Medicine, Huashan Hospital, Fudan University; Sports Medicine Institute of Fudan University, Shanghai 200040, China.

*Correspondence: dongyu.dy@163.com; biochenjun@fudan.edu.cn; sjfeng13@fudan.edu.cn

^#^These authors contributed equally to this work.


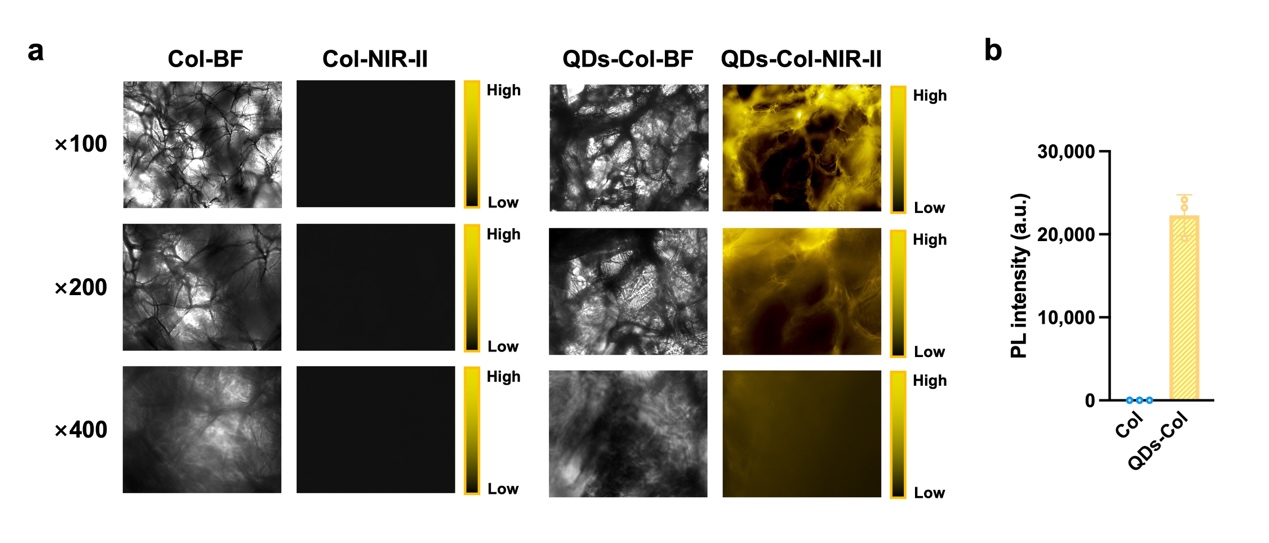


**Supplementary Figure 1.** **Labeling efficiency of QDs-labeled collagen observed under microscope.** (a) BF and NIR-II images were captured at magnifications of ×100, ×200, and ×400. The fluorescence intensity, indicated by the color bars adjacent to the NIR-II images, ranged from low (black) to high (yellow). (b) The PL intensity between collagen and QDs-labeled collagen was quantified.


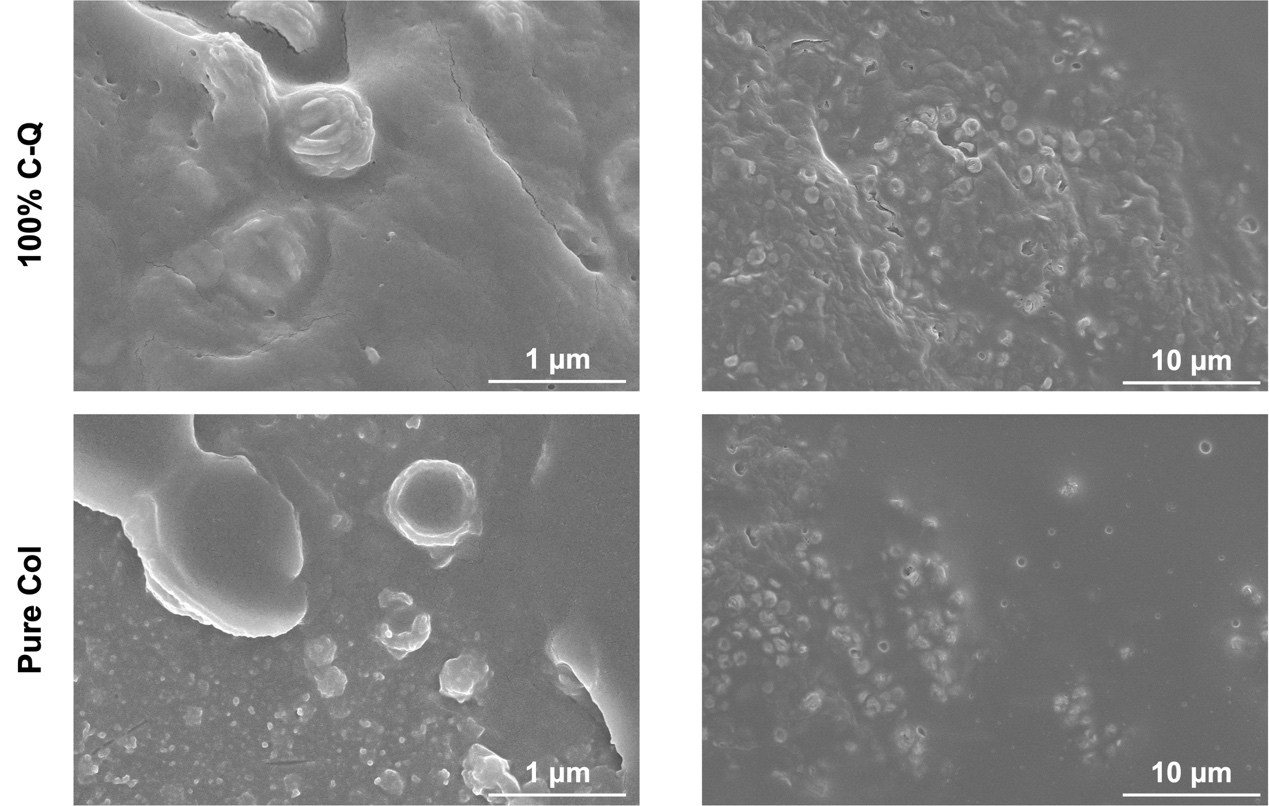


**Supplementary Figure 2. SEM images of pure collagen and 100% C-Q.**


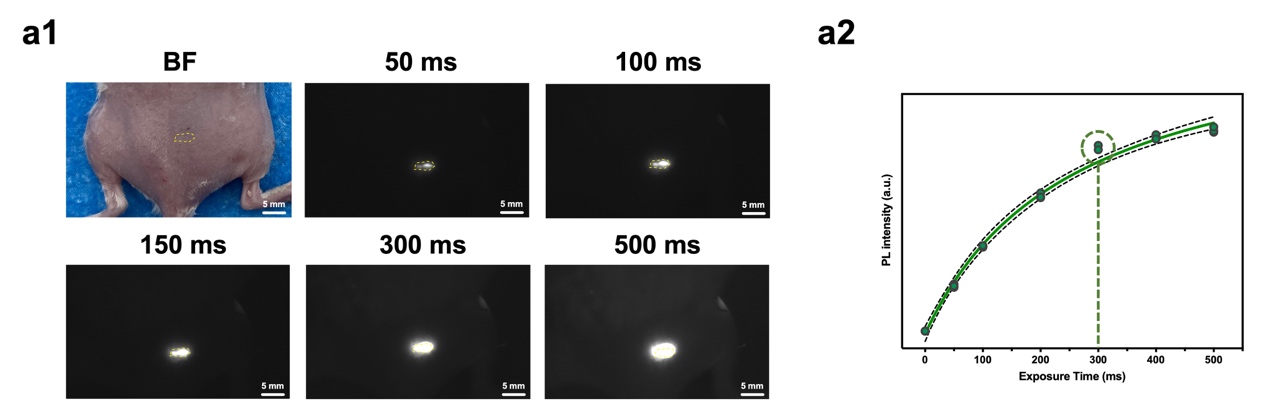


**Supplementary Figure 3. Representative NIR-II images of subcutaneous implant group and corresponding PL intensity curves under varying exposure conditions.** BF and NIR-II images of the (a1) subcutaneous implant group captured at exposure time ranging from 50 to 500 ms. (a2) Nonlinear relationship between PL intensity and exposure time. The dashed green line and circle indicated the optimal exposure baseline.


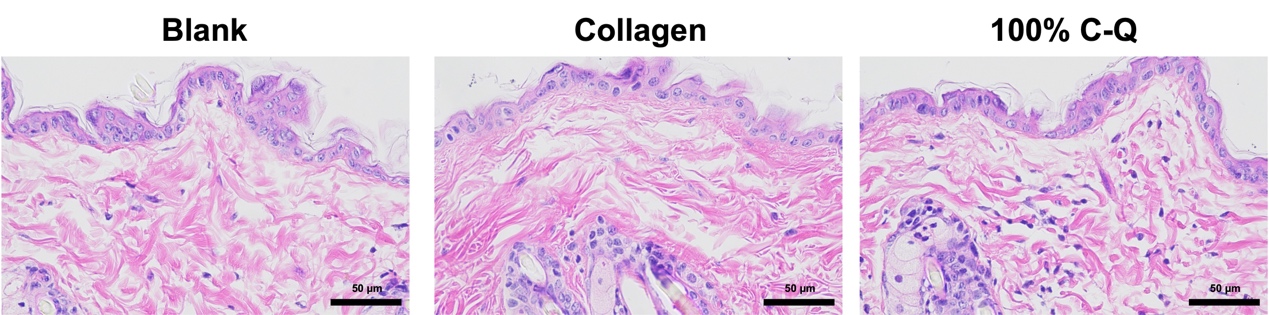


**Supplementary Figure 4. Tissue reaction after collagen implantation.** Hematoxylin and eosin (H&E) staining of subcutaneous tissue harvested 35 days after post implantation from the mice with collagen and 100% C-Q implantation, compared with a blank group.


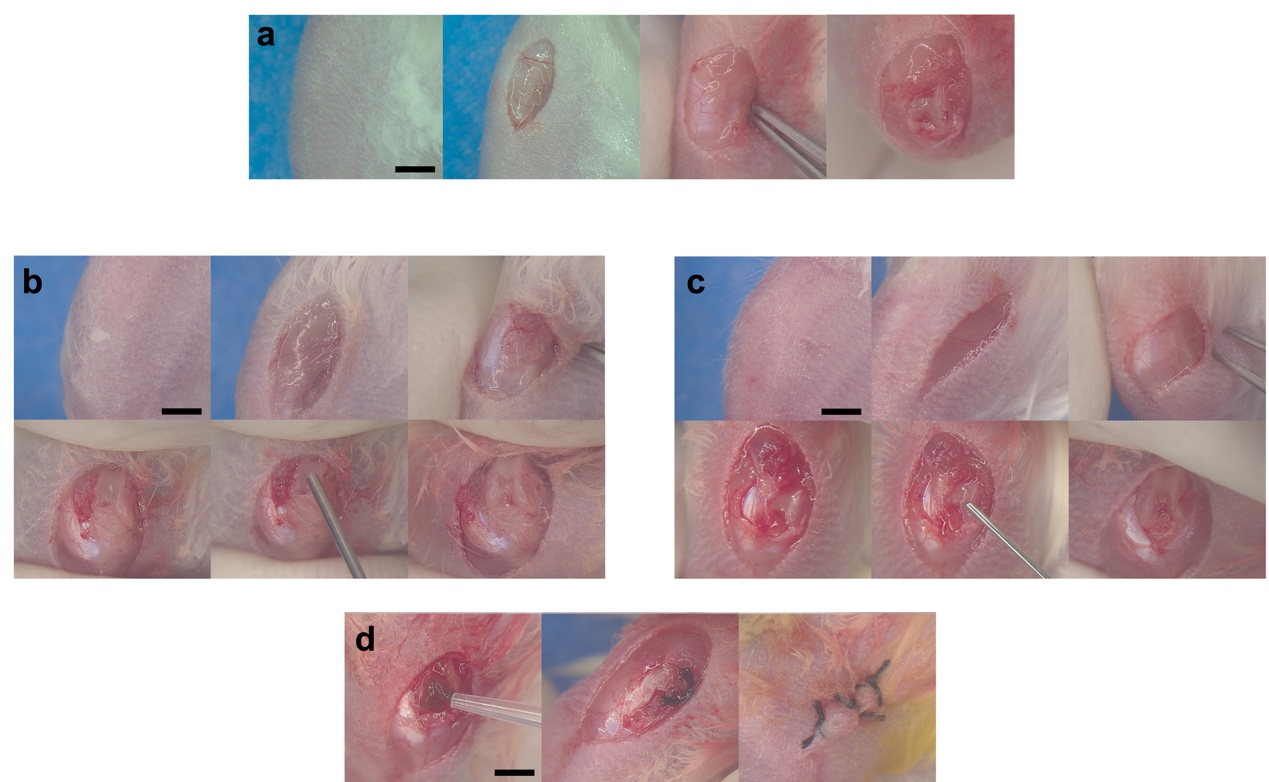


**Supplementary Figure 5.** **Surgical procedure of different groups and collagen implantation.** (a) Sham group. (b) CSD group. (c) Minor group. (d) Collagen implantation and suture. Scale bar, 4 mm.


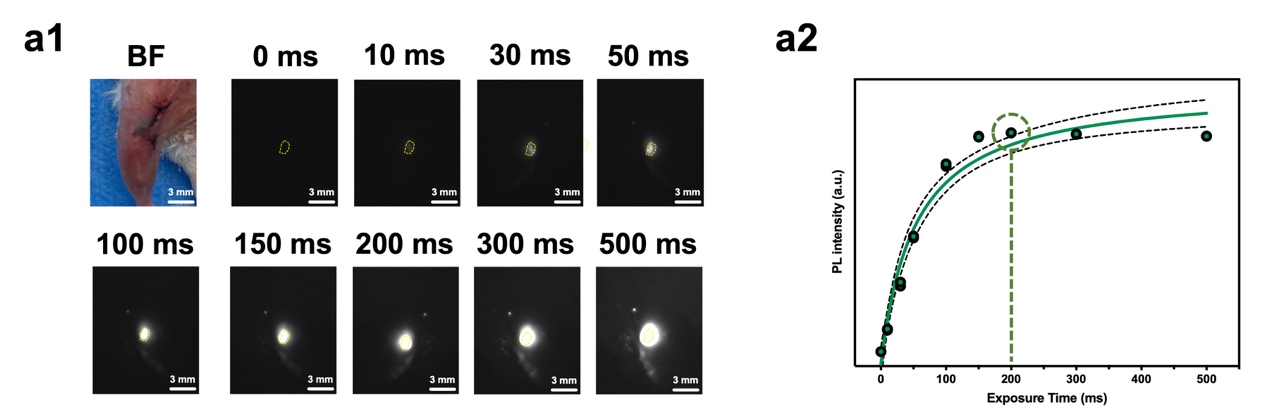


**Supplementary Figure 6. Representative NIR-II images of cartilage defect group and corresponding PL intensity curves under varying exposure conditions.** BF and NIR-II images of the (a1) cartilage defect group captured at exposure time ranging from 0 to 500 ms. (a2) Nonlinear relationship between PL intensity and exposure time. The dashed green line and circle indicated the optimal exposure baseline.


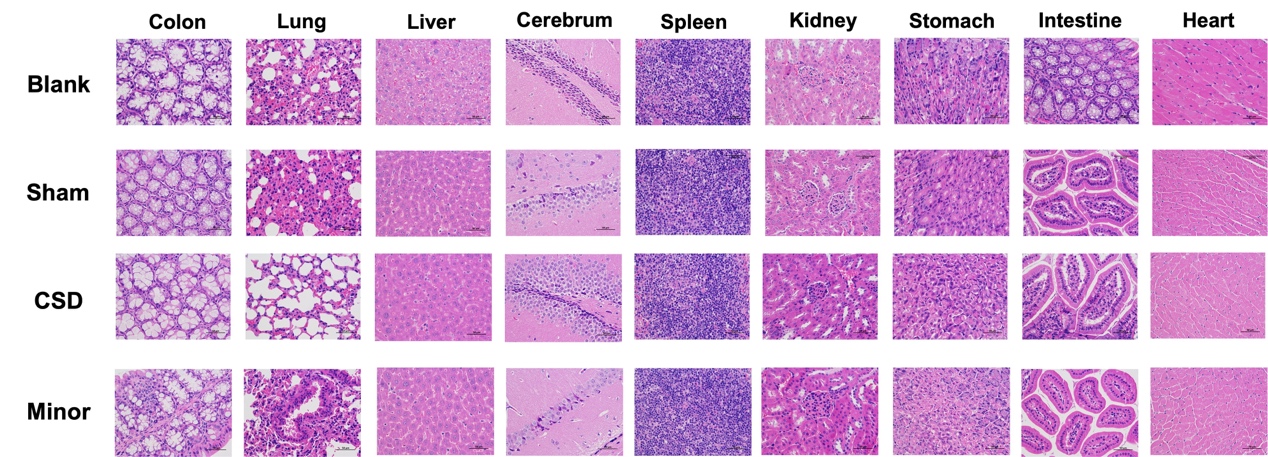


**Supplementary Figure 7. Biosafety verification of QDs-labeled collagen.** H&E staining of major organs including colon, lung, liver, cerebrum, spleen, kidney, stomach, intestine and heart from healthy mice (Blank), Sham group, CSD group and Minor group. Magnification: ×400.
